# Prognostic factors for incomplete response in thyroid microcarcinoma: an analysis of initial response to therapy in 517 patients

**DOI:** 10.20945/2359-3997000000341

**Published:** 2021-03-19

**Authors:** Fernanda Nascimento Faro, Vivian Roberta Ferreira Simões, Gustavo Piech Ricardo, Cristal Peters Cabral, Karina de Cássia Braga Ribeiro, Nilza Maria Scalissi, Adriano Namo Cury, Marília Martins Marone, Rosália do Prado Padovani, Carolina Ferraz

**Affiliations:** 1 Unidade de Doenças da Tireoide Divisão de Endocrinologia Departamento de Medicina São Paulo SP Brasil Unidade de Doenças da Tireoide, Divisão de Endocrinologia, Departamento de Medicina, Irmandade da Santa Casa de Misericórdia de São Paulo, São Paulo, SP, Brasil; 2 Faculdade de Ciências Médicas da Santa Casa de São Paulo Departamento de Saúde Coletiva São Paulo SP Brasil Departamento de Saúde Coletiva, Faculdade de Ciências Médicas da Santa Casa de São Paulo, São Paulo, SP, Brasil; 3 Faculdade de Ciências Médicas da Santa Casa de São Paulo São Paulo SP Brasil Faculdade de Ciências Médicas da Santa Casa de São Paulo, São Paulo, SP, Brasil; 4 Irmandade da Santa Casa de Misericórdia de São Paulo Serviço de Medicina Nuclear São Paulo SP Brasil Serviço de Medicina Nuclear, Irmandade da Santa Casa de Misericórdia de São Paulo, São Paulo, SP, Brasil

**Keywords:** Thyroid neoplasms, thyroid microcarcinoma, prognostic factors, active surveillance, multifocality

## Abstract

**Objective:**

Although thyroid microcarcinoma (TMC) usually has a favorable prognosis, some patients present a higher risk of disease recurrence or persistence. Thus, we aimed at identifying possible risk factors associated with an incomplete response to therapy in TMC.

**Subjects and methods:**

This was a retrospective study of 517 patients with TMC treated with total thyroidectomy, with or without radioactive iodine (RAI) therapy, reclassified after 1.1 ± 0.4 years according to the response to treatment into “favorable” (excellent/indeterminate) or “unfavorable” (biochemical/structural incomplete) responses. We evaluated participants' age, sex, tumor size, histological variants, multifocality, presence of vascular/lymphatic/perineural invasion, extrathyroidal extension, metastatic lymph nodes (LN), and distant metastasis. The effect of RAI therapy on the response range was analyzed in a given subgroup.

**Results:**

The mean age observed was 46.4 ± 12.0 years, and 89.7% were female. We noted 97.5% with papillary carcinoma, 27.8% with multifocality and 11.2% with LN metastasis. Although the majority of patients had a low risk of recurrence/persistence (78%), 75% were submitted to RAI therapy. Incomplete response (20.7%) was associated with multifocality (p=0.041; OR=1.619) and metastatic LN (p=0.041; OR=1.868). These variables were strongly correlated (p=0.000; OR=3.283). No cut-off of tumor size was identified as a predictor of incomplete response by the receiver operating curve analysis. RAI treatment did not influence the response of patients with multifocality or LN metastasis.

**Conclusion:**

Multifocality and LN metastasis are independent risk factors for incomplete response in TMC patients and are strongly correlated. Additional RAI therapy was not associated with a more favorable response in these subgroups.

## INTRODUCTION

Thyroid cancer is the most common endocrine neoplasm, and its incidence is on a significant rise in the last 30 years (1). This is mainly due to a greater screening of thyroid microcarcinoma (TMC), defined as a tumor measuring 1 cm or less in its largest diameter, and has contributed to almost 50% of new cases (2,3).

Moreover, mortality from thyroid cancer remains low and stable, suggesting that TMC is, in most cases, an indolent tumor, detected incidentally on ultrasound examination (3,4). In addition, TMC is present in 5 - 36% of individuals in autopsy studies due to death from non-thyroidal causes and is an anatomopathological finding in 10% of thyroid glands removed due to benign disease (5).

Therefore, with its low risk of persistence and/or recurrence and a mortality rate of less than 1% (6), active surveillance has been proposed as an appropriate therapeutic strategy for low-risk patients, with outcomes similar to that of surgical treatment (7).

Nevertheless, some patients with TMC show a higher risk of disease recurrence and may require a more aggressive approach (8). Currently, some factors have been suggested as possible indicators of worse outcomes; however, there is still debate in the literature about their prognostic value.

The majority of studies involving risk factors for TMC focus on parameters that predispose to the development of lymph node (LN) metastasis at diagnosis (9–12). However, according to the guidelines of the American Thyroid Association (ATA) 2015 (13), a good way to assess a patient's prognosis is to classify them correctly according to the risk of recurrence or persistence of the disease and on the type of response after 6–24 months of the initial treatment (14), considering not only evidence of structural disease but also the values of serum thyroglobulin (Tg) and anti-Tg antibody (TgAb) levels.

The ATA's “response to therapy restaging system” (13) classifies patients into four categories: excellent, indeterminate, biochemical incomplete and structural incomplete response. To the best of our knowledge, there is no available evidence on the prognostic factors of TMC for response range using the dynamic risk stratification proposed by the ATA. Thus, the aim of this study was to identify possible risk factors associated with an initial incomplete response in patients with TMC.

## SUBJECTS AND METHODS

### Patients and study design

**Figure 1:**
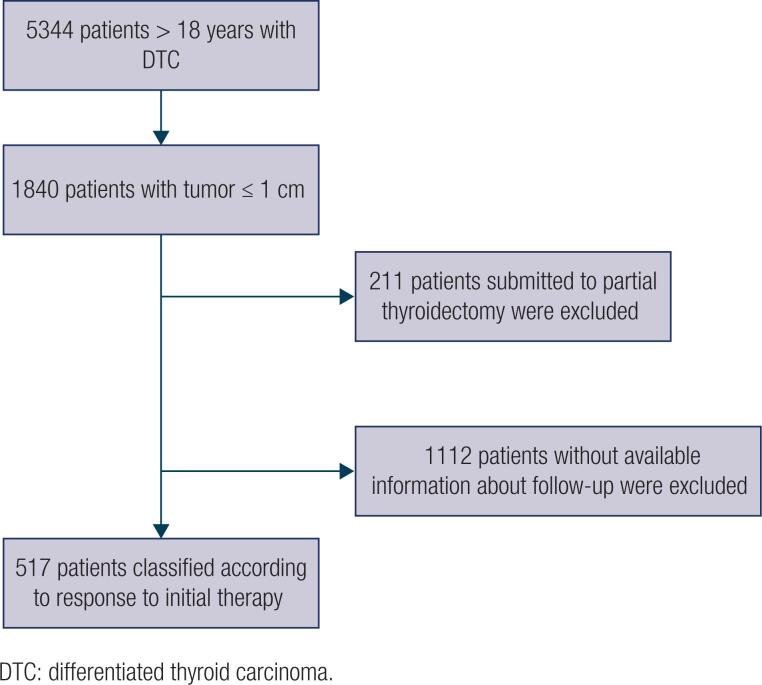
Flowchart of patients with thyroid microcarcinoma included in the retrospective cohort study.

A total of 5344 patients who underwent thyroid surgery for malignancy between 1972 and 2015 compose the database of DTC patients from the Nuclear Medicine Service, Nuclimagem (Figure 1). Nuclimagem is a nuclear medicine service that provides specialized care to patients in *Santa Casa de Misericórdia de São Paulo*. It receives patients from Santa Casa and all other regions of the state, referred by the Sao Paulo State Health Secretary to undergo treatments or examinations. For this reason, a long follow-up period was not possible for most patients.

Patients who were diagnosed with TMC (a total of 1840) were selected. TMC was defined as a thyroid tumor 1 cm or less in diameter, in accordance with the WHO classification (15). Therefore, only adults (> 18 years) who underwent total thyroidectomy, with or without additional RAI therapy, with complete follow-up data from 6 to 24 months after initial treatment were included in this study. Patients who underwent partial thyroidectomy (211 patients) or those with incomplete follow-up data (the measurement of Tg, TgAb, whole-body scintigraphy [WBS], or neck ultrasound [US]) were excluded (Figure 1). Due to the wide variation in TgAb assays in recent years, we also opted to remove patients with positive TgAb in our study to avoid possible errors in assessing the type of treatment response.

Subsequently, we had available information to classify 517 patients according to the ATA dynamic risk stratification using the response to therapy restaging system (13). The patients were evaluated retrospectively. They were also followed-up for a mean of 1.1 ± 0.4 years (from 6 to 24 months), with a physical examination, thyroid ultrasonography, measurement of serum Tg and TgAb levels, and with or without a diagnostic WBS.

Patients were also stratified using TNM staging according to the 8th AJCC/UICC (16) to predict disease mortality and according to the ATA initial risk stratification system (13) to predict the risk of disease recurrence and/or persistence (13).

### Outcomes

We reevaluated patients 6-24 months after initial treatment (first assessment after ablation or first assessment after surgery in those patients not subjected to RAI treatment) by measuring their levels of thyroid-stimulating hormone (TSH), Tg, TgAb and stimulated thyroglobulin (sTg), and by performing a neck US, with or without a WBS. Overall, 125 patients did not undergo sTg. In these cases, the suppressed Tg level was used as an alternative.

Clinical status was assessed for each of the 517 patients and was defined by classifying patients' response to initial treatment as excellent, indeterminate, biochemical incomplete and structural incomplete responses, according to the 2015 ATA classification (13). To enable statistical analysis, patients with excellent and indeterminate responses were classified as having a “favorable response”, while those with biochemical or structural incomplete responses were classified as “unfavorable response”. These groups were pooled into a single incomplete response group, given the need for similar treatment (suppressive levothyroxine therapy) and closer follow-up.

Excellent response was considered if the imaging findings were negative and the patient had either a suppressed Tg level of < 0.2 ng/mL or a sTg level of < 1.0 ng/mL if remnant RAI ablation was performed, or Tg level of < 0.2 ng/mL or a sTg level of < 2.0 ng/mL without RAI ablation. The indeterminate response indicated non-specific findings on imaging studies, faint uptake in the thyroid bed on RAI scanning, and a non-stimulated detectable Tg level of ≥ 0.2 ng/mL but < 1.0 ng/mL with RAI ablation or 0.2 - 5.0 ng/dL without RAI ablation or a sTg detectable level ≥ 1.0 but < 10 ng/mL with RAI ablation or ≥ 2.0 but < 10 ng/dL without RAI ablation. Biochemically incomplete response was reported if the imaging findings were negative and the patient had either a sTg level of ≥ 1.0 ng/mL with RAI ablation or ≥ 5.0 ng/dL without RAI ablation or a sTg level of ≥ 10 ng/mL. A structural incomplete response was indicated if there was structural or functional evidence of disease despite Tg levels (13).

### Study's variables

The following variables were evaluated as risk factors to an “unfavorable response”: age at diagnosis (< 55 and ≥ 55 years), gender, tumor size at the largest diameter, histology (papillary or follicular carcinoma), variants of papillary carcinoma (classic, follicular, and others), multifocality, presence of vascular/lymphatic/perineural invasion, extrathyroidal extension, presence of metastatic LN or distant metastasis. Multifocality was present when two or more tumor foci were found in the thyroid lobes. The presence of metastatic LN was considered when LN involvement was greater than 0.2 cm.

We also evaluated the additive effect of RAI therapy on the response range.

### Assays

The analyses of sTg at ablation and TgAb were performed in the same laboratory, and the levels were assessed using the immulite thyroglobulin assay. This is a first-generation Tg assay with a functional sensitivity (FS) of 1.0 ng/mL and a lower limit of detection of 0.2 ng/mL.

For patients assessed for response to initial treatment until 2015, sTg was used because second-generation Tg assays were not available in our laboratory. After 2015, patients' response to initial treatment was evaluated by measuring the sTg levels because as second-generation Tg assays started becoming available in our institution. Thereafter, Tg levels were assessed using the chemiluminescent assay (Access Thyroglobulin Assay; Beckman Coulter) with an FS of 0.1 ng/mL.

TgAb was measured by a chemiluminescent assay (Immulite 2000 [Diagnostic Products Corporation; reference value of up to 40 IU/mL] or ARCHITET TgAb [Abbott Laboratories; reference value of up to 4.11.

### Imaging methods

Neck US was performed by experienced ultrasonographers from our Institution using a high-resolution color Doppler US apparatus with a 7.5-MHz linear transducer. Diagnostic or post-therapeutic 131I-WBS was performed using a one-head – camera (Apex SPX 4000; Elscint Italia, Milan, Italy) with a high-energy collimator and a sensitivity of 160 cpm/mCi. The scan speed was 10 cm/min, with a total count of 100,000 cpm minimum.

### Statistical analysis

Statistical analyses were performed using SPSS version 20.0. Categorical variables were expressed as absolute and relative frequencies and were compared using the chi-square or Fisher's exact test where appropriate. Continuous variables were expressed as mean and standard deviation. Univariate analysis was performed to investigate the association between incomplete responses and clinical variables. A multivariate logistic regression model was performed to investigate independent risk factors significantly related to incomplete response, and the odds ratios (ORs) for this analysis were calculated for variables that were significant in the univariate analyses. Statistical significance was considered at p values < 0.05.

A receiver operating characteristic (ROC) curve was used to identify the best cut-off of tumor size at the largest diameter to predict incomplete response. It was based on the values of sensitivity and specificity and on the calculation of the area under the curve of tests. The Youden index was used to estimate the best cut-off, based on the fact that the highest index represents the best balance point between sensibility and specificity.

To evaluate the effect of RAI activities on the initial response to therapy, we exclusively analyzed the subgroup of patients with significant findings in multivariate analyses, considering the hypothesis that this particular group would more likely benefit from additional treatment. We compared the response range in patients who had received RAI therapy after surgery and those who underwent total thyroidectomy without RAI therapy.

The study was approved by the local research Ethics Committee (CCAE: 28945420.5.0000.5479).

## RESULTS

We retrospectively evaluated 517 patients diagnosed with TMC. The mean age at diagnosis was 46.4 ± 12.0 years, and 89.7% were female. The majority of the patients (97.5%) had papillary carcinoma. The mean tumor size was 0.6 ± 0.2 cm. Multifocality was present in 27.8% of the sample, and 11.2% had lymph node metastasis at the time of diagnosis.

The AJCC/TNM Staging System, 8^th^ Edition, stratified 97.5% of the patients as stage I. According to the ATA Initial Risk Stratification, most patients had a low risk of disease recurrence or persistence (78%). Despite this, 75% of patients underwent RAI therapy as an adjuvant treatment. Additional histological features are shown in Table 1.

**Table 1:** Baseline characteristics of TMC (thyroid microcarcinoma) patients according to the American Thyroid Association (ATA) risk stratification (13)

Characteristics		n	%
Age (years) at diagnosis		
	< 55	396	76.6
	≥ 55	121	23.4
Female gender		464	89.7
Histology			
	Papillary	502	97.5
	Follicular	13	2.5
Histology variant of papillary carcinoma			
	Classic	141	50.4
	Follicular	120	42.8
	Others	19	6.8
	Multifocality	143	27.8
Extrathyroidal invasion			
	No	133	77.8
	Microscopic invasion	24	14.0
	Macroscopic invasion	14	8.2
	Vascular invasion	34	6.6
	Lymphatic invasion	27	5.2
	Perineural invasion	3	0.6
	Metastatic lymph nodes	60	11.6
	Distant metastasis	1	0.2
AJCC/TNM staging system			
	I	504	97.5
	II	9	1.7
	III	4	0.8
ATA initial risk stratification system			
	Low risk	403	78.0
	Intermediate risk	100	19.3
	High risk	14	2.7
RAI therapy		387	75.0
Response classification to initial therapy			
	Excellent	331	64.0
	Indeterminate	79	15.3
	Biochemical incomplete	42	8.1
	Structural incomplete	65	12.6

AJCC: American Joint Committee on Cancer, 8^th^ edition; ATA, American Thyroid Association; RAI: radioactive iodine.

After a mean of 1.1 ± 0.4 years (0.5 to 2 years) of the initial treatment, patients were reclassified in relation to their response to initial therapy. An incomplete response was observed in 20.7% of cases. In univariate analysis, multifocality (p = 0.012; OR = 1.774; 95% CI [1.113 - 2.784]) and LN metastasis at diagnosis (p = 0.010; OR = 2.121; 95% CI [1.181 - 3.808]) were significantly associated with incomplete response (Table 2). Multivariate analysis confirmed that they were independent risk factors for incomplete response (Table 3). In addition, these two variables were found to be strongly correlated with each other (p = 0.000; OR = 3.283; 95% CI [1.895 - 5.687]) (Table 4).

**Table 2:** Univariate analysis of factors associated with incomplete response 6-24 months after initial therapy

Characteristics		Excellent/Indeterminate	Incomplete	P value	Test	OR*	95% CI for OR	
							Lower	Upper
Age (years) at diagnosis	< 55	318	78	0.310	Chi-square			
	≥ 55	92	29					
Gender	Female	368	96	0.991	Chi-square			
	Male	42	11					
Histology	Papillary	398	104	0.738	Fisher's Exact			
	Follicular	10	3					
Histology variant of papillary carcinoma	Classic	112	29	0.286	Fisher's Exact			
	Follicular	90	30					
	Others	12	7					
Multifocality	No	306	67	0.012	Chi-square	*1		
	Yes	103	40			1.774	1.113	2.784
Extrathyroidal invasion	No	79	54		Chi-square			
	Microscopic invasion	17	7	0.236				
	Macroscopic invasion	6	8					
Vascular invasion	No	384	98	0.393	Chi-square			
	Yes	25	9					
Lymphatic invasion	No	388	100	0.498	Chi-square			
	Yes	20	7					
Perineural invasion	No	407	106	0.503	Fisher's Exact			
	Yes	2	1					
Metastatic lymph nodes	No	369	87	0.010	Chi-square	*1		
	Yes	40	20			2.121	1.181	3.808

* OR reference: category 1; OR: odds ratio; CI: confidence interval; significant difference at p ≤ 0.05.

**Table 3:** Factors associated with incomplete response by multivariate analysis

Characteristics		OR	95% CI for OR		P value
			Lower	Upper	
Multifocality	Yes	1.619	1.019	2.571	0.041
Presence of metastatic lymph nodes	Yes	1.868	1.024	3.406	0.041
Constant		0.207			0.000

* OR: odds ratio; CI: confidence interval; significant difference at p ≤ 0.05.

All factors that were significant in the univariate analysis were included in the multivariate analysis (Table 2): the presence of metastatic lymph nodes and multifocality.

**Table 4:** Association between multifocality and presence of metastatic lymph nodes

Metastatic lymph nodes	Multifocality		P value	Test	OR*	95% CI for OR	
	No	Yes				Lower	Upper
No	344	112	0.000	Chi-square	1*	1.895	5.687
Yes	29	31			3.283		

* OR reference: category 1; OR: odds ratio; CI: confidence interval; significant difference at p ≤ 0.05.

**Figure 2:**
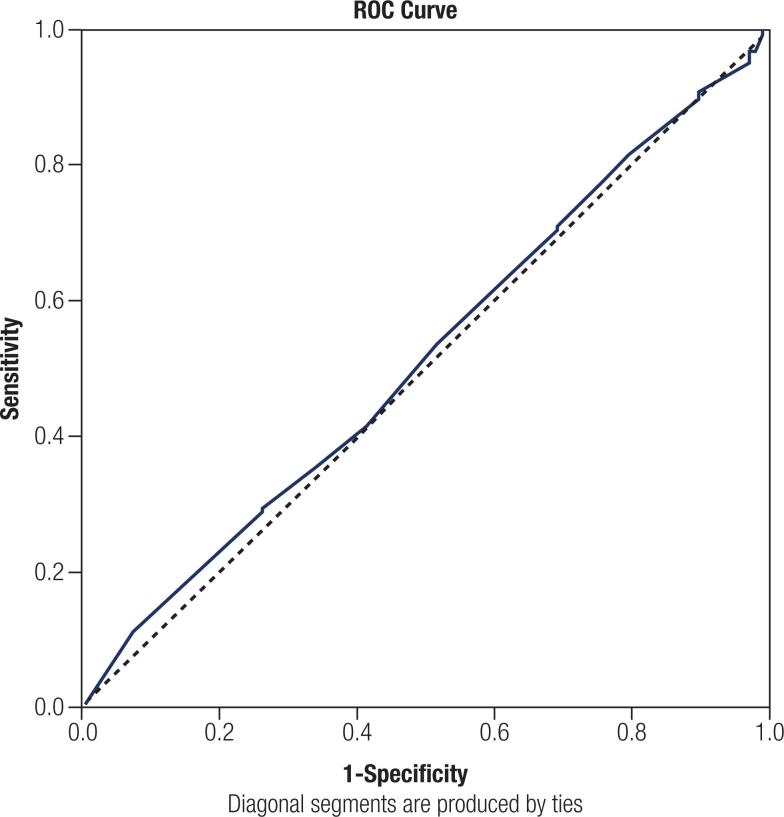
ROC (Receiver Operating Characteristic) curve used to identify the best cut-off of tumor size at the largest diameter to predict incomplete response. Area under the curve = 0.516; Highest Youden Index = 0.037.

To identify the best cut-off of tumor size at the largest diameter to predict an incomplete response, ROC curve analysis was performed. However, as demonstrated in Figure 2, owing to the similarity between the curve and the diagonal, no point of the curve resulted in a significant difference in the response range.

Regarding the effect of RAI therapy on the initial response, no significant association was observed between RAI treatment and response range in patients with the multifocal disease (p = 0.467) (Table 5). Furthermore, similar results were found in the subgroup of patients with metastatic LN at the time of diagnosis (Table 5). In this analysis, although Fisher's test showed a p-value of 0.043, the univariate analysis demonstrated no significant association (OR = 10.7; 95% CI [0.5-195.9]). Therefore, additional treatment with RAI therapy was not related to a better outcome, even when analyzed exclusively in the subgroups of patients with a higher risk of an unfavorable response.

**Table 5:** Association between RAI therapy and response range in the subgroup of patients with presence of multifocality and metastatic lymph nodes

RAI therapy		Response range		P value	Test	OR*	95% CI for OR	
		Excellent/Indeterminate	Incomplete				Lower	Upper
Multifocality								
	No	18	5	0.467	Chi-square	*1		
	Yes	85	35			1.482	0.510	4.305
Metastatic lymph nodes								
	No	8	0	0.043	Fisher's Exact	*1		
	Yes	32	20			10.723	0.587	195.918

* OR reference: category 1; OR: odds ratio; CI: confidence interval; RAI: radioactive iodine; significant difference at p . 0.05.

Pagano and cols. (31) and Dicks and cols. (32) suggested the addition of the constant 0.5 in all cells as a method of correction to eliminate the zero-value cell and enable the use of odds ratio calculation algorithms. Although this result seems to contradict Fisher's exact test, the zero-value cell and the large difference between the number of patients who used the radioiodine compared to those who did not highlight the test's weakness. As the p-value of Fisher's exact test is close to the level of significance adopted (5%) and the OR was not significant, we conclude that there is no relationship between the use of RAI therapy and the response range for patients with lymph node metastasis.

## DISCUSSION

Due to the evidence that TMC generally has an indolent course and a low risk of recurrence and/or persistence, the ATA considers the possibility of active surveillance instead of immediate surgery in patients with low-risk TMC (13). However, the identification of prognostic factors is important in deciding which patients are more suitable for active surveillance and for other therapeutic strategies.

In accordance with Ito and cols. (17) and Tuttle and cols. (18), patients with LN involvement or signs of extrathyroidal extension (signs or symptoms of invasion of the recurrent laryngeal nerve or trachea) are not eligible for active surveillance. Moreover, Brito and cols. (19) divided patients into ideal, appropriate, and inappropriate candidates for active surveillance. In these categories, the authors suggest that the presence of extrathyroidal extension, multifocality, LN metastasis and other factors should be considered.

To our knowledge, this is the first study to correlate risk factors with the response range of dynamic ATA risk stratification in patients with TMC. In our study, 20.7% of the patients with TMC presented with an unfavorable response (biochemical and structural incomplete responses) after the initial treatment. This is higher than that reported by Mazzaferri and cols. (6), who found locoregional and distant recurrences of TMC in 5.9% and 1.5%, respectively. This is probably because we used evidence of both structural and biochemical disease to classify patients' response to therapy. In addition, our data were from a tertiary service where patients are referred to obtain specialized care.

LN metastasis (p = 0.041; OR = 1.868; 95% CI [1.024 - 3.406]) and multifocality (p = 0.041; OR = 1.619; 95% CI [1.019-2.571]) were independently associated with incomplete response. An interesting descriptive and meta-analysis study of TMC dating from 2008 also showed that multifocality (p = 0.001; OR = 0.174; 95% CI [0.105 - 0.290]) and lymph node metastasis (p = 0.001; OR = 0.213; 95% CI [0.136 - 0.336]) at diagnosis were positively associated with a higher risk of recurrence, especially in patients younger than 45 years (20). In the same way, tumor size, gender, vascular and extrathyroidal invasion did not play a significant role as impact factor for recurrence.

The majority of studies involving risk factors for TMC focus on variables that predispose to the development of LN metastasis at diagnosis as the main characteristic associated with a worse prognosis (9–12). We demonstrated that metastatic LNs and multifocality were strongly associated with each other. Karatzas and cols. (21), in a retrospective study of 539 patients with papillary carcinoma of whom 57.7% had TMC, also demonstrated this association. Guo and cols. (22) revealed in a study of 329 patients that TMC with 2, 3, or 4 foci had a higher risk of LN metastasis. Kaliszewski and cols. (11) showed that the presence of multifocal or bilateral tumors was a significant predictor of LN metastasis in a retrospective study of 177 patients with TMC and metastatic LN. A meta-analysis of 14 studies and 4573 patients found that a higher risk of central LN metastases was associated with multifocality, but not with bilateral tumors (23). All these findings lead us to consider the possibility of LN metastases in thyroid glands with the presence of multifocal TMC over time, thus suggesting that these patients are not eligible for active surveillance only, as suggested first by Brito and cols. (19), or will need an earlier rescue surgery.

Although the impact of LN metastasis is well established, there are ongoing debates about multifocality being a cause of more aggressive behavior and a higher risk of disease recurrence and persistence in TMC. Again, multifocality is almost always related to LN metastasis than is assessed as an independent risk factor for a worse prognosis. Hay and cols. (24) reported that multifocality and LN metastasis at diagnosis increased the risk for nodal involvement (mean follow-up of 17 years), with 11% of multifocal tumors exhibiting higher recurrence/persistence compared with only 4% of unifocal tumors. In a Korean study that evaluated clinical and pathological characteristics and prognostic factors in 527 TMC patients, multifocality and sex were identified as risk factors for metastatic LN in young patients (< 45 years), whereas multifocality and LN metastasis were identified in older patients (> 45 years) (25). We could not find any association between incomplete response and age or sex. On the other hand, some studies did not find a correlation of a worse prognosis of multicentricity, especially if the nodules were in the same lobe or smaller than 1 cm (26,27).

It is also mentioned that tumor size plays a role as a prognostic factor (20), but in our findings, no cut-off of tumor size was considered a good predictor for an incomplete response. Although there are no studies comparing tumor size and response range to our knowledge, several studies have evaluated the relationship between tumor size and the presence of metastatic lymph nodes. Jin and cols. (28) found that a tumor size of 5 mm or higher was an independent predictor of central LN metastases, while tumors larger than 7 mm were predictors of central or lateral LN metastases. A meta-analysis of 19 studies and 8345 TMC patients also demonstrated that central LN metastases were associated with tumor size > 5 mm (29). Lee and cols. (26) and Xu and cols. (30), found that a tumor size of > 7 mm was associated with more aggressive features. Nevertheless, the authors agree that the smaller the tumor, the lower the probability of LN metastasis. However, the association between tumor size and response range is still not established and requires further study.

RAI therapy is not indicated for most patients with TMC because of its low risk of tumor recurrence (13). In our study, the rate of RAI therapy was high (75%) because the sample comprised of patients with TMC diagnosed since the 1970s when RAI therapy was indicated for all patients, independent of the risk. According to the ATA 2015 guidelines, RAI therapy is only indicated for high-risk patients and can be recommended in intermediate-risk patients, as cases of TMC with the presence of LN metastases (five or more lymph nodes affected with a size of > 0.2 cm) (13).

Therefore, our aim was to assess whether these patients who evolved with an incomplete response due to multifocality or LN metastasis would benefit from additional RAI treatment. However, they did not show a better response range than patients in the same group without RAI therapy. Similar results were found in a study of 900 TMC patients with a follow-up of 60 years that observed higher tumor recurrence in patients with multifocal TMC or LN metastasis at diagnosis, with RAI remnant ablation not significantly reducing tumor recurrence rates (24).

Unfortunately, long-term follow-up of the patients was not performed in our study. Moreover, surgery and follow-up of patients were not carried out at the same center, and we consider these as limitations to our study, although RAI was administered in the same laboratory, using the same protocols. As mentioned, the Nuclear Medicine Division of Santa Casa de São Paulo is a tertiary center that receives patients from several other centers to undergo RAI therapy. Consequently, surgical ability, the extent of surgery (with or without lymphadenectomy), histological details (as the number of metastatic lymph nodes), and follow-up protocols specific to each service could be considered as confounding factors.

Therefore, we conclude that although patients with TMC are considered low risk and evolve well, there is a particular group of patients (those showing multifocality and LN metastasis at the time of diagnosis) which can evolve with an incomplete response and thereby benefit from surgical treatment without complementary radioiodine treatment, as the latter did not show any additional benefit for this group of patients in our study. Long-term follow-up studies are strongly recommended to corroborate our findings.
